# Outcomes in Cochrane Systematic Reviews Addressing Four Common Eye Conditions: An Evaluation of Completeness and Comparability

**DOI:** 10.1371/journal.pone.0109400

**Published:** 2014-10-16

**Authors:** Ian J. Saldanha, Kay Dickersin, Xue Wang, Tianjing Li

**Affiliations:** Department of Epidemiology, Johns Hopkins Bloomberg School of Public Health, Baltimore, Maryland, United States of America; University of Newcastle, Australia, Australia

## Abstract

**Introduction:**

Choice of outcomes is critical for clinical trialists and systematic reviewers. It is currently unclear how systematic reviewers choose and pre-specify outcomes for systematic reviews. Our objective was to assess the completeness of pre-specification and comparability of outcomes in all Cochrane reviews addressing four common eye conditions.

**Methods:**

We examined protocols for all Cochrane reviews as of June 2013 that addressed glaucoma, cataract, age-related macular degeneration (AMD), and diabetic retinopathy (DR). We assessed completeness and comparability for each outcome that was named in ≥25% of protocols on those topics. We defined a completely-specified outcome as including information about five elements: *domain*, *specific measurement*, *specific metric*, *method of aggregation*, and *time-points*. For each domain, we assessed comparability in how individual elements were specified across protocols.

**Results:**

We identified 57 protocols addressing glaucoma (22), cataract (16), AMD (15), and DR (4). We assessed completeness and comparability for five outcome domains: quality-of-life, visual acuity, intraocular pressure, disease progression, and contrast sensitivity. Overall, these five outcome domains appeared 145 times (instances). Only 15/145 instances (10.3%) were completely specified (all five elements) (median = three elements per outcome). Primary outcomes were more completely specified than non-primary (median = four versus two elements). Quality-of-life was least completely specified (median = one element). Due to largely incomplete outcome pre-specification, conclusive assessment of comparability in outcome usage across the various protocols per condition was not possible.

**Discussion:**

Outcome pre-specification was largely incomplete; we encourage systematic reviewers to consider all five elements. This will indicate the importance of complete specification to clinical trialists, on whose work systematic reviewers depend, and will indirectly encourage comparable outcome choice to reviewers undertaking related research questions. Complete pre-specification could improve efficiency and reduce bias in data abstraction and analysis during a systematic review. Ultimately, more completely specified and comparable outcomes could make systematic reviews more useful to decision-makers.

## Introduction

In clinical trials, an outcome is an event or measure in study participants that is used to assess the effectiveness and/or safety of the intervention being studied [1]. Choosing relevant outcomes is a critical early step in the design of clinical trials and systematic reviews for a number of reasons [2]. In clinical trials, expected effect sizes on critical outcomes are used to determine sample size [3]. In addition, there is general agreement that by pre-specifying the primary and secondary outcomes and limiting the number of statistical analyses, clinical trialists reduce the likelihood of Type I error (i.e., finding a statistically significant treatment effect just by chance, in the absence of a true treatment effect) and outcome reporting bias (i.e., selectively reporting outcomes based on the strength and/or direction of the findings). Although satisfactory solutions have not yet been developed, there is growing recognition that these issues also apply to systematic reviews [4]–[5]. Indeed, the Cochrane Collaboration recommends that systematic reviewers limit the number of and pre-specify all outcomes for their systematic review [6]–[7].

**Figure 1:**
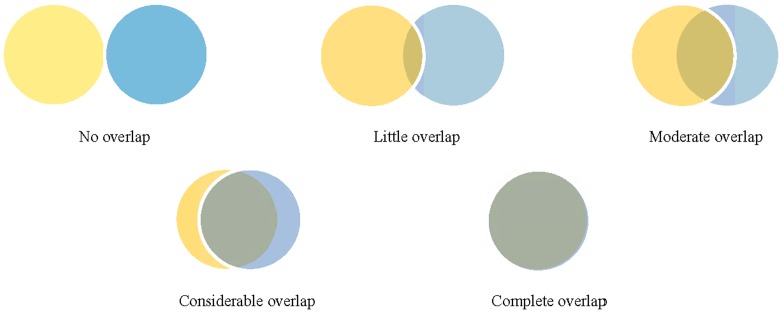
Examples of extent of overlap of possible outcome domains chosen by clinical trialists and systematic reviewers. Yellow - Outcomes chosen by clinical trialists. Blue - Outcomes chosen by systematic reviewers. Grey - Outcomes chosen by BOTH clinical trialists and systematic reviewers.

The process of conducting a systematic review of intervention effectiveness begins with formulating a research question, and then, finding and synthesizing the evidence from studies that address the question. In formulating the question, the systematic reviewer defines the population, intervention, comparison, and outcomes (PICO) to be examined. Studies that address the review question, typically clinical trials, should be broadly similar on the population, intervention and comparison groups, but frequently report different outcomes from those chosen by the systematic reviewer. Clinical trialists typically measure numerous outcomes, sometimes in the hundreds [8]. It is likely that these outcomes are different from those chosen by the systematic reviewer; overlap of the chosen outcomes can vary from none to complete (Figure 1). In many cases, the primary outcome of interest to the systematic reviewers may not have been an outcome of interest to the clinical trialists [9], or may not be reported clearly or consistently in the clinical trial reports or associated documents [10]. Systematic reviewers thus face an important decision: should they choose outcomes to be examined based on what they believe to be important outcomes (“systematic review author judgment”) or based on what they know is reported in the relevant clinical trials (“clinical trialist judgment”)?

How systematic reviewers choose outcomes and pre-specify them in systematic review protocols is currently unclear. One view is that, unlike clinical trialists, systematic reviewers should not base outcome choice on sample size/power calculations and Type I error rates. Instead, the objective of medical research should be to draw conclusions based on all sources of available evidence [11]. Systematic reviews, which are often used to inform clinical practice guidelines and policy, could and even should include all the outcomes that patients, clinicians, and policy-makers need to know about. Systematic reviews also allow elucidation of existing research gaps in a given field [12], for example, when outcomes are not examined in trials and should be.

In our view, regardless of who chooses the outcomes to be assessed in a systematic review and how those outcomes are chosen, all outcomes need to be specified completely and clearly if they are to be of use to decision-makers.

The objective of our study was to assess the completeness of pre-specification and comparability of outcomes in all Cochrane reviews addressing four common eye conditions. Our purpose is not to hold systematic review protocols to a standard that may not have been described at the time they were published, rather it is to initiate a discussion on important questions for systematic reviewers: how should systematic reviewers choose outcomes to address in the review; how should these outcomes be reported (i.e., which elements are necessary for complete reporting) by systematic reviewers; and if outcomes are pre-specified in systematic review protocols, should these protocols be formally updated with amendments to reflect changing outcome specification?

## Methods

### Review protocols examined

The Cochrane Collaboration publishes and archives all its systematic review protocols, completed reviews, and updates in *The Cochrane Database of Systematic Reviews*. Protocols for systematic reviews, hereafter referred to as ‘protocols’, were eligible for our study if they were published by the Cochrane Eyes and Vision Group (CEVG) in *The Cochrane Database of Systematic Reviews* in or before June 2013 (Issue 6), and if they addressed any of the following four eye conditions: glaucoma, age-related macular degeneration (AMD), cataract, and diabetic retinopathy (DR). We selected these four conditions because of their high disease burden across populations and the range of interventions addressing them [13]. For each eligible review, we identified the oldest available protocol and, when no protocol could be found for a review, we contacted CEVG editors and review authors via email to ask whether they had a copy. When these efforts were not successful, we used the most recent version of the completed review in place of the protocol.

### Five elements of a completely specified outcome

**Figure 2:**
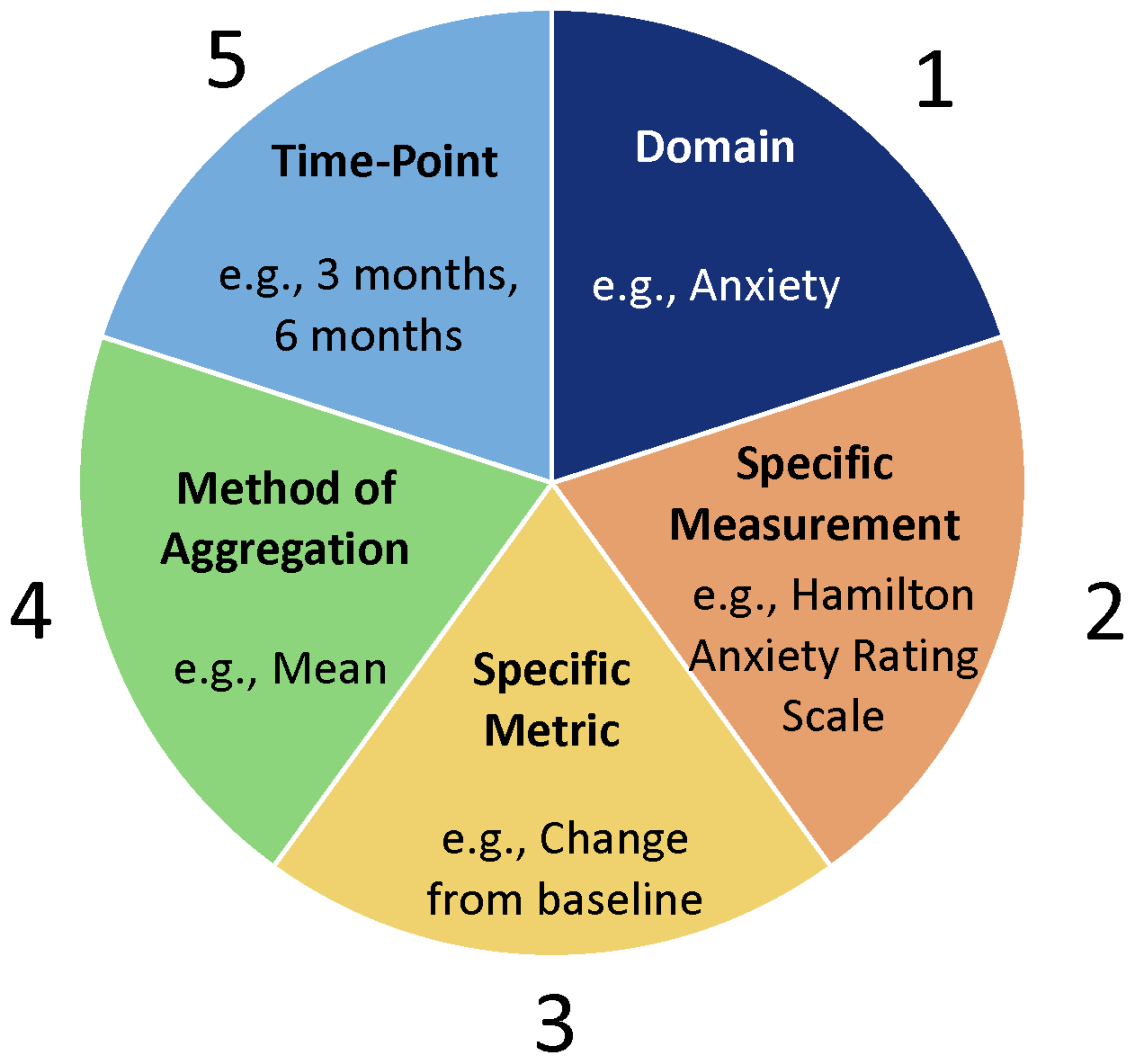
Five elements of a completely specified outcome, with anxiety as an example.

We used an outcome definition that includes five elements: (1) the *domain* or outcome title (e.g., visual acuity); (2) the *specific measurement* or technique/instrument used to make the measurement (e.g., Snellen chart); (3) the *specific metric* or format of the outcome data from each participant that will be used for analysis (e.g., value at a time-point, change from baseline); (4) the *method of aggregation* or how data from each group will be summarized (e.g., mean, percent/proportion); and (5) the *time-points* that will be used for analysis (e.g., 3 months) (Figure 2). Whereas Zarin et al. specify these same elements [8], they define the first four elements and consider time-points related to each of those four.

### Selecting outcome domains for data extraction

Before beginning data extraction, one investigator (IS) identified all outcome domains in the Methods sections of included protocols. We then selected for data extraction those outcome domains appearing in at least 25% of eligible protocols. Then, for those eligible protocols with published completed reviews, we compared the Methods section of the protocol with the Methods section of the most recent version of the corresponding completed review, noting any differences in the specified outcome domains. We did this step to evaluate whether focusing on the protocols, some of which were published a while ago, would mean that we were assessing a different set of outcome domains than those currently being evaluated by the review authors.

### Data extraction

We designed, tested, and finalized a data extraction form using Google Forms^©^. Two investigators (IS and XW) extracted data independently and resolved discrepancies through consensus or discussion with a third author (TL). We extracted data about the eye condition and year of publication of each protocol. We extracted from the Methods section the following data pertaining to each eligible outcome: type of outcome (primary, non-primary, or unclear [if not specified]) and each of the five outcome elements described earlier. For element 2, we extracted all specific measurements that were specified, or classified the specific measurement as unclear (if not specified). We classified element 3 (specific metric) into one or more of the following categories: (i) value at a time-point, (ii) time-to-event, (iii) change from baseline, and (iv) unclear (if not specified). We classified element 4 (method of aggregation) into one or more of the following categories: (i) mean, (ii) median, (iii) percent/proportion, (iv) absolute number, and (v) unclear (if not specified). For element 5, we extracted all time-points that were specified, or classified the time-points as unclear (if not specified).

### Data analysis

We assessed the extent of completeness using the number of elements specified out of five possible, and considered an outcome specified in the Methods section as “complete” if all five elements were specified. For each outcome, we calculated median, interquartile range (IQR), and proportion of outcome elements specified. We performed Kruskal-Wallis tests for nonparametric comparisons of medians and distributions of extent of completeness by condition addressed, year of protocol publication, type of outcome, and outcome domain.

We assessed the frequency and comparability of outcome elements (i.e., similarity of categories for each element) for elements 3 and 4 across protocols addressing each of the four eye conditions. Protocols could specify more than one category for a given element. Comparability was therefore assessed as the distribution of those categories across protocols. As an example, if one protocol specified visual acuity at a time-point as well as change in visual acuity from baseline, we counted both categories for specific metric (element 3). In another example, protocols addressing cataract and assessing the outcome of visual acuity were considered to be comparable in method of aggregation (element 4) if they all specified mean or all specified median or both. However, they would not be comparable in element 4 if some specified mean and others specified median.

Statistical significance was defined at the 5% level. All data were analyzed using STATA^©^ version 12 (College Station, TX).

## Results

### Characteristics of protocols examined

Our search identified 57 eligible systematic reviews (Table 1). We were able to find protocols for 54 reviews (94.7%), and used the Methods section of completed reviews for the remaining three (5.3%). An updated protocol was published for one of the 54 protocols. Glaucoma was the most frequently addressed condition (22 protocols), followed by cataract (16 protocols), AMD (15 protocols), and DR (4 protocols). Approximately half of the protocols (29/57; 50.9%) were published between 2006 and 2010. Thirty-four protocols were associated with a completed review, the most recent version of which was published a median of five (IQR 4–8, range 0–15) years after publication of the protocol.

**Table 1:** Number of protocols and outcome domains by condition, year published, and whether specified as primary outcome.

Characteristic	Number (%) of protocols	Number (%) of outcomes
All	57^1^ (100)	145^2^ (100)
Condition addressed		
Glaucoma	22 (38.6)	51 (35.2)
Cataract	16 (28.1)	35 (24.1)
Age-related macular degeneration (AMD)	15 (26.3)	47 (32.4)
Diabetic retinopathy (DR)	4 (7.0)	12 (8.3)
Year of protocol publication		
2000 or earlier	6 (10.5)	13 (9.0)
2001 to 2005	15 (26.3)	37 (25.5)
2006 to 2010	29 (50.9)	76 (52.4)
2011 or later	7 (12.3)	19 (13.1)
Type of outcomes domain specified	Not applicable	
Outcomes specified as primary		48 (33.1)
Outcomes specified as non-primary		88 (60.7)
Type of outcome unclear		9 (6.2)

1 54 protocols and 3 completed reviews; One protocol did not include any of the outcome domains selected for detailed data extraction.

2 139/145 of the outcomes were described in the 54 protocols.

### Outcome domains used in protocols

We examined five outcome domains named in at least 25% of the eligible protocols (Table 2): quality-of-life (47/57 protocols; 82.5%), visual acuity (47/57; 82.5%), intraocular pressure (21/57; 36.8%), disease progression (15/57; 26.3%), and contrast sensitivity (15/57; 26.3%). One protocol did not name any of these five outcome domains. For most completed systematic reviews (30/34; 88.2%), these five outcome domains were similar to what was named in their corresponding protocols. Compared to their protocols, two completed systematic reviews dropped quality-of-life while one completed review added it. One completed systematic review dropped contrast sensitivity.

**Table 2:** Completeness (number of completely-specified elements out of five possible) by outcome domain.

Characteristic	Number (%) of protocols	Median (IQR) number of completely-specified elements per outcome	p-value
All	57^1^ (100)	3.0 (2.0–4.0)	-
Outcome domain			
Quality-of-life	47 (82.5)	1.0 (1.0–2.0)	0.0001
Visual acuity	47 (82.5)	3.0 (2.0–4.0)	
Intraocular pressure	21 (36.8)	4.0 (3.0–4.0)	
Disease progression	15 (26.3)	3.0 (2.0–4.0)	
Contrast sensitivity	15 (26.3)	2.0 (1.0–3.0)	

1 One protocol did not include any of the outcome domains selected for detailed data extraction.

### Completeness of outcome pre-specification

Across the 57 protocols, the five most frequent outcome domains appeared 145 times (‘instances’); however, only 15/145 instances (10.3%) involved complete pre-specification (i.e., where all five elements of the outcome were specified). Overall, a median of three (IQR 2–4) elements were specified per outcome (Table 3). Extent of completeness was not statistically significantly different by condition. Completeness of outcome specification may be better in protocols published later compared to earlier, (median of three [IQR 2–4] elements specified in 2006–2010 versus one [IQR 1–3] in 2000 or earlier), although the difference was not statistically significant (p = 0.1635).

**Table 3:** Completeness (number of completely-specified elements out of five possible) by type of protocol/outcome.

Characteristic	Median (IQR) number of completely specified elements per outcome	p-value
All^1^	3.0 (2.0–4.0)	NA
Condition addressed		
Glaucoma	3.0 (2.0–4.0)	0.1218
Cataract	3.0 (2.0–4.0)	
Age-related macular degeneration (AMD)	2.0 (1.0–3.0)	
Diabetic retinopathy (DR)	3.0 (1.5–4.0)	
Year of protocol publication		
2000 or earlier	1.0 (1.0–3.0)	0.1635
2001 to 2005	2.0 (2.0–4.0)	
2006 to 2010	3.0 (2.0–4.0)	
2011 or later	2.0 (2.0–3.0)	
Type of outcome domain specified		
Outcomes specified as primary	4.0 (3.0–4.0)	0.0001
Outcomes specified as non-primary	2.0 (1.0–3.0)	
Type of outcome not specified	1.0 (1.0–2.0)	

1 54 protocols and 3 completed reviews; Median 3.0 (2.0–4.0) for outcomes in the 54 protocols and 1.5 (1.0–2.0) for outcomes in the 3 reviews (p = 0.0627); One protocol did not include any of the outcome domains selected for detailed data extraction.

**Figure 3:**
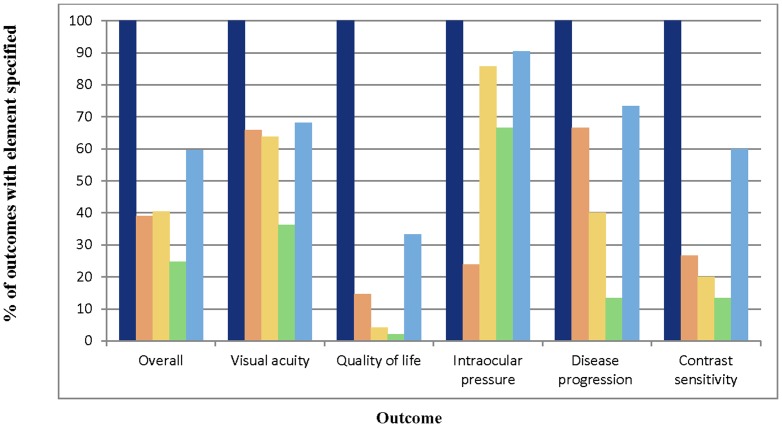
Completeness of specification of outcome elements, by outcome. Navy blue - Domain. Orange – Specific measurement. Yellow – Specific metric. Green – Method of aggregation. Blue – Time-point(s).

**Figure 4:**
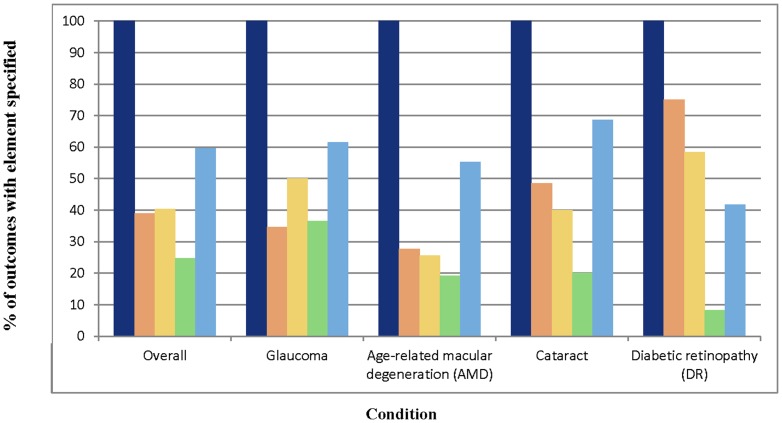
Completeness of specification of outcome elements, by condition. Navy blue - Domain. Orange – Specific measurement. Yellow – Specific metric. Green – Method of aggregation. Sky blue – Time-point(s).

Fifty-four of 57 protocols (94.7%) specified at least one primary outcome. Among the five outcome domains evaluated in our study, at least one was a primary outcome in 48/57 (84.2%) protocols. Extent of completeness appeared to differ by outcome type, with primary outcomes being most completely specified and outcomes with type unclear being least completely specified (median four versus one respectively, p = 0.0001). Intraocular pressure was the most completely specified outcome in our sample, with a median of four (IQR 3–4) elements specified (Table 2). Quality-of-life was least completely specified, with a median of one (IQR 1–2) element specified. The patterns of completeness of individual elements were similar across outcomes (Figure 3). Method of aggregation was specified least often, while domain and time-points were specified more often than other elements. The completeness of individual elements for the quality-of-life outcome was less than for other outcomes, overall. Although intraocular pressure was the most completely specified outcome, only 24% of protocols assessing it specified the specific measurement. Patterns of completeness of individual outcome elements also appeared to be similar across conditions, except for outcomes in DR protocols, where there were only four protocols and so the percentages are unlikely to be reliable (Figure 4).

Table 4 provides some examples of incomplete specification of outcomes in our sample of systematic reviews.

**Table 4:** Examples of incomplete outcome pre-specification.

Exact text from methods section of protocol	Number of completely-specified elements (out of five possible)
*“The primary outcome for the review will be visual acuity.”*	1
*"When available quality of life data will be described for those with operated and unoperated cataract."*	1
*“Postoperative visual acuity”*	1
*“Quality of life”*	1
*“Contrast sensitivity”*	1
*“Vision-related quality of life at one year"*	2
*“Mean IOP"*	2

### Comparability of outcome elements

Table 5 shows the distribution of specific metrics (element 3) and methods of aggregation (element 4) across instances of usage of outcome domain, by condition. The specific metric was unclear for large proportions of individual instances (often as high as 100% for the 16 instances of usage of quality-of-life in protocols addressing glaucoma and for the four instances of usage of contrast sensitivity in protocols addressing cataract). For instances where the specific metric was specified, the most frequent specific metrics were ‘value at a time-point’ and ‘change from baseline’.

**Table 5:** Frequency of categories of specific metric (element 3) and method of aggregation (element 4) across instances of usage of outcome domains by condition.

Condition/Outcome domain (Number of protocols/Number of instances)	Categories of specific metric(element 3)(% of instances)				Categories of method of aggregation(element 4)(% of instances)			
	Value at a time-point	Time-to-event	Change from baseline	Unclear	Mean	Percent/proportion	Absolute number	Unclear
Glaucoma (22 protocols)								
Quality-of-life (16 instances)	-	-	-	100	-	-	-	100
Visual acuity (13 instances)	31	-	31	46	8	39	-	54
Intraocular pressure (20 instances)	55	10	25	15	50	10	-	40
Disease progression (2 instances)	-	50	50	-	-	-	-	100
Cataract (16 protocols)								
Quality-of-life (12 instances)	17	-	-	83	-	8	-	92
Visual acuity (15 instances)	53	-	20	33	-	33	-	67
Intraocular pressure (1 instance)	100	-	-	-	100	100	-	-
Disease progression (3 instances)	-	-	33	67	-	-	-	100
Contrast sensitivity (4 instances)	-	-	-	100	-	-	-	100
Age-related macular degeneration (15 protocols)								
Quality-of-life (15 instances)	-	-	-	100	-	-	-	100
Visual acuity (15 instances)	47	-	33	40	20	13	13	60
Disease progression (6 instances)	-	-	-	100	-	-	17	83
Contrast sensitivity (11 instances)	18	-	9	73	18	18	-	82
Diabetic retinopathy (4 protocols)								
Quality-of-life (4 instances)	-	-	-	100	-	-	-	100
Visual acuity (4 instances)	100	-	25	-	-	-	-	100
Disease progression (4 instances)	50	25	-	25	25	-	-	75

Notes:

• If there was no instance of usage of a certain outcome domain across all reviews addressing a given condition, the above table does not include a row for that outcome domain for that condition.

• Percentages are row percentages (adding up individual categories within an element). Percentages sometimes total more than 100% for an element because some protocols used more than one category for that element.

• No reviews used “median” as a method of aggregation (element 4).

The method of aggregation was unclear for large proportions of individual instances (often as high as 100% for the 16 instances of usage of quality-of-life in protocols addressing glaucoma and for the four instances of usage of visual acuity in protocols addressing DR). For instances where the method of aggregation was specified, the most frequent methods of aggregation were ‘mean’ and ‘percent/proportion’.

## Discussion

### Summary of main findings

We have shown that, if outcome pre-specification in systematic review protocols is judged using recommended standards for clinical trials, then it is largely incomplete. Although completeness appears to have improved somewhat over time, on average, only three of five standard elements of an outcome were pre-specified. Due to largely incomplete outcome pre-specification, a conclusive assessment of comparability in outcome elements across the various protocols per condition was not possible. However, we observed variation in specific metrics and methods of aggregation.

### Completeness of outcome pre-specification

There are some reasons that might explain why outcomes were not completely specified in our study of systematic review protocols. First, although we believe complete specification of all five elements is necessary for a number of reasons, the idea is new to the systematic review community. This is demonstrated by the fact that the Cochrane Handbook states only that the name of the outcome (equivalent to *domain* [element 1]), type of scale (equivalent to *specific measurement* [element 2]), and timing of measurement (equivalent to *time-points* [element 5]) must be pre-specified^6^; and there is no explicit mention of pre-specification of *specific metric* (element 3) or *method of aggregation* (element 4). Indeed, elements 1 and 5 were the most often-specified elements in our sample of protocols, though element 2 was frequently not specified (70% of the time) (Figure 3).

Another possible explanation for incomplete pre-specification of outcomes is that choice of outcomes could be influenced by the findings of (and outcomes examined in) the clinical trials that would be included in the review. We did not assess the outcomes examined at the level of the clinical trials to determine the likelihood that this occurred, but suggest that doing so may contribute to a better understanding of how review outcomes are chosen. Are they chosen because systematic reviewers consider them the most important outcomes to examine, because they are the outcomes that have been examined in clinical trials, or both? If the review outcomes were chosen purely because they were the outcomes that have been reported in clinical trials, this is troubling because of the possibility of “meta-bias”. We know, for example, that outcomes reported in clinical trials could have been selectively reported because of desirable or undesirable findings [14]–[15]. By pre-specifying in the protocol the outcomes to be examined in the review, systematic reviewers minimize the potential for bias [5], [16], and reassure readers that the choice of outcomes was not influenced by the results of individual clinical trials. That said, systematic reviewers are usually familiar with their field and *a priori* aware of potentially eligible clinical trials and/or how the outcome in question is frequently measured. Complete pre-specification also could improve efficiency in data abstraction and analysis during a systematic review.

Systematic reviewers may also anticipate potential variation in outcomes across included clinical trials, and may allow for this by pre-specifying the elements of the outcome domain of interest in broad rather than specific terms (e.g., “visual acuity” versus “change in visual acuity from baseline to 1 year, as measured using a Snellen chart”). If such variation is suspected, systematic reviewers could explicitly state that all variations of a given element(s) will be included. This could minimize the occurrence of what Page et al. refer to as “selective inclusion” in systematic reviews [5].

We assume that primary outcomes for both clinical trials and systematic reviews are chosen based on perceived clinical importance and/or importance to patients; and that they are usually measured and reported more thoroughly than non-primary outcomes [17]. Not surprisingly, in our study, primary outcomes were more completely specified than other outcome types. Our estimate of 94.7% protocols pre-specifying a primary outcome is somewhat higher than the 88% that has been reported as pre-specified in clinical trial protocols [18], and this could be related to the fact that we were examining protocols entered into software that requests the domain names of the pre-specified outcomes.

In our study, the most incompletely pre-specified outcome was quality-of-life, a key patient-important outcome. This finding is concordant with other studies that have found that outcome reporting in clinical trials is a bigger problem for patient-important outcomes than other types of outcomes [19]–[20]. Further, when patient-important outcomes are not primary outcomes in clinical trials, the likelihood that reporting is complete is further reduced [20]. Our study aimed to evaluate the completeness and comparability of all outcomes, both patient-important and not.

Our recommendation is that systematic reviewers should engage in discussion about and strongly consider pre-specifying all five elements of each outcome they wish to examine. When explicit pre-specification of all five elements of a given outcome is not possible, for example when all possible options for a given outcome element are not known or are too numerous, the systematic reviewers should enumerate all known acceptable options for each element and explicitly state that all options for that element would be accepted, or provide rationale for why it is impossible to completely pre-specify an element.

The Preferred Reporting Items for Systematic Reviews and Meta-analyses Protocols (PRISMA-P) is currently under development [21]. We hope that the availability of reporting guidelines (including details about outcome specification) will improve the completeness of specification of outcomes. Assuming that the Cochrane Collaboration recognizes the importance of completeness of pre-specification, there are some possible ways to ensure that review authors are aware of the five elements of a completely specified outcome. First, editorial teams at Cochrane Review Groups (CRGs) should make all review authors aware of the five outcome elements early in the process (no later than the protocol development stage). Second, peer reviewers should be directed to consider whether the outcomes are completely pre-specified and not likely to have been chosen based on the strength and direction of the findings for those outcomes. Third, the Cochrane Handbook and other systematic review guidance materials, in addition to training workshops and other educational avenues, should incorporate explicit descriptions of all five outcome elements. Other organizations producing guidance on systematic review methodology (e.g., Agency for Healthcare Research and Quality [AHRQ], the Centre for Reviews and Dissemination [CRD]) should also incorporate descriptions of the five outcome elements in their guidance materials.

Organizations such as the Cochrane Collaboration suggest limiting the number of outcomes examined in a systematic review [6]. However, in order to evaluate whether the effect of an intervention persists over time, an otherwise identical outcome (i.e., identical in the other four elements) is often measured at a number of time-points. For the purpose of counting the number of outcomes measured, we recommend that these repeated measurements be counted as one outcome regardless of the number of time-points at which the outcome is assessed.

### Comparability of outcome elements

In the era of evidence-based medicine, decision-makers in healthcare (e.g., patients, clinicians, and policy-makers) increasingly rely on systematic reviews. It is important that decision-makers have access to high quality and up-to-date individual systematic reviews as well as are able to compare results across systematic reviews. Cochrane “overviews” (Cochrane reviews which compile evidence from related reviews of interventions into a single accessible and usable document) [6], and network meta-analyses (analyses of three or more interventions for a given condition in one meta-analysis) [22]–[23] are examples of formal comparisons across systematic reviews. To better feed into these formal comparisons and clinical practice guidelines, the elements of outcomes used in the various systematic reviews addressing a given condition should be comparable. In our study, the largely incomplete pre-specification of outcomes in protocols restricted our ability to assess comparability in outcome elements across protocols. In cases where the various elements were specified, however, we observed variation in specific metrics and methods of aggregation. An example of such variation is: one protocol pre-specified that the outcome domain of visual acuity would be measured as mean change in visual acuity (number of letters) from baseline to one year, while another protocol pre-specified that visual acuity would be measured as percent of participants with improvement in visual acuity of at least three letters at one year. While both protocols specified the same outcome domain at the same time-point, differences in the specific metric (mean change versus value at a time-point) and method of aggregation (mean versus percent) would preclude a direct comparison of the visual acuity results.

Efforts to promote comparability of outcomes across related clinical trials have led to the creation of core outcome measures within research fields [24]–[26]. One such effort is the Core Outcome Measures in Effectiveness Trials (COMET) Initiative [27], whose investigators have produced guidance on methods for identifying core outcome sets [28]. Because the issue of comparability of outcomes across systematic reviews is complex, we recommend that researchers within a field (e.g., systematic reviewers, Cochrane review group editors, clinical trialists) and patients consider developing comparable outcomes across systematic reviews, adding to a core list over time as appropriate.

There are pros and cons of establishing comparability in outcomes across reviews, however. Increased comparability will likely facilitate formal comparisons across systematic reviews and development of clinical practice guidelines. In addition, decision-makers would be better able to compare more directly the effectiveness of treatment options. For example, hundreds of measurement scales (*specific measurements*) have been used to assess mental status in schizophrenia [29] and quality-of-life [30], making comparability across clinical trials very challenging. Finally, use of comparable outcomes could discourage authors from ‘cherry-picking’ outcomes to be used in their studies [31].

On the other hand, comparability across reviews is not always possible or desirable. Limiting outcomes to those used by previous researchers risks excluding an outcome that is in fact important, or authors may be compelled to include an outcome that they do not consider important. Additionally, it might not be possible to identify *a priori* all relevant outcomes and outcome elements for a rapidly evolving field or for a field with a large number of relevant outcomes.

### Availability of protocols and amendments to protocols

We were unable to obtain 3/57 (5.3%) protocols associated with our sample of Cochrane reviews. This poses a concern for investigators conducting methodological research in systematic reviews, and for users of systematic reviews generally. Although we do not believe that relying on the Methods sections of three completed Cochrane reviews in the cases where we could not find the protocols is likely to have influenced our findings, we believe that all protocols and previous versions of completed systematic reviews should be made available to researchers. Furthermore, an updated protocol was published for only one of the protocols we examined. The Cochrane Collaboration should consider keeping all protocols up-to-date by publishing updated versions of protocols or publishing protocol amendments for all its reviews. In this way, Cochrane review protocols would be formally amended in the same way that clinical trial protocols are amended and made available, providing an accessible audit trail. This practice will facilitate Cochrane's contribution of its protocols and updates to PROSPERO [32]–[33], an international database of prospectively registered systematic reviews.

Our focus on Cochrane reviews is both a strength and a limitation. Assuming that Cochrane reviews are among the most rigorously conducted and reported systematic reviews [34]–[35], it is likely that completeness and comparability of outcomes are higher in our sample of reviews than in other reviews. It would be useful to know how others producing systematic reviews (e.g., AHRQ, CRD, independent authors) choose and describe outcomes in their systematic reviews.

As discussed, we did not examine the individual clinical trials examined by each Cochrane review in our sample to learn more about the source of non-comparability in outcome elements. Nor did we test for empirical evidence of outcome reporting bias on the part of the systematic reviewers. Because our assessments of completeness and comparability were based on what was reported in the protocols (and some completed reviews), it is possible that our findings were a consequence of unsatisfactory reporting and that the rationale for the outcomes chosen could not be determined without asking the systematic review authors directly.

Our study should be replicated in other disease areas and on a larger scale to assess the applicability of our findings to other fields. Although we have compared the outcomes pre-specified in the protocol with what is in the corresponding completed review's Methods section, a next step would be to compare the outcomes in the Methods with those in the Results section. This would allow a confirmation of the potential bias by systematic reviewers that has been demonstrated by Kirkham et al. using a cohort of Cochrane reviews [36] and by various investigators studying this issue in clinical trials [14], [17], [37]–[38].

## Conclusions

We recommend that systematic review authors strongly consider pre-specifying all outcomes of interest using the five elements of a completely specified outcome (domain, specific measurement, specific metric, method of aggregation, and time-points), amending the protocol formally, as needed. We further suggest that researchers and other stakeholders, such as patients, carefully consider the pros and cons of establishing comparability in outcomes across systematic reviews addressing a given condition.
